# An introduction to the fundamentals of root systems research in cultivated plants

**DOI:** 10.1093/aob/mcag155

**Published:** 2026-06-04

**Authors:** Dylan H Jones, Akshay B Tawale, Hannah M Schneider

**Affiliations:** Department of Physiology and Cell Biology, Leibniz Institute of Plant Genetics & Crop Plant Research (IPK) OT Gatersleben, Corrensstr 3, Seeland 06466, Germany; Department of Physiology and Cell Biology, Leibniz Institute of Plant Genetics & Crop Plant Research (IPK) OT Gatersleben, Corrensstr 3, Seeland 06466, Germany; Department of Physiology and Cell Biology, Leibniz Institute of Plant Genetics & Crop Plant Research (IPK) OT Gatersleben, Corrensstr 3, Seeland 06466, Germany; Division of Root Sciences, Department of Crop Science, Georg-August-University Goettingen, Goettingen 37075, Germany

**Keywords:** Roots, anatomy, architecture, root types, physiology, plasticity

## Abstract

**Background:**

Although often overlooked due to being hidden below ground, plant roots are diverse in function and structure, and are fundamental to plant growth and future crop improvement due to their role in resource acquisition, anchorage, interaction with soil organisms and, in some cases, providing the crop harvestable product itself. Root systems are dynamic, expressing physiological variation between species, cultivars, environments and even within the continuum of a single root, at each level contributing uniquely to plant and ecosystem processes.

**Scope:**

Designed as an educational review, this article provides a structured and accessible overview of the complex biology of roots in cultivated plants. We establish a foundational framework by defining different types of root systems, root classes and specialized root types. Serving as a primer for students, educators and researchers expanding their expertise, we review how variation in root traits affects plant growth and performance under stress conditions, and briefly introduce how some of these traits are genetically regulated. Finally, we summarize specific root research areas that have shown recent promise, offering a roadmap for further study.

**Conclusions:**

Gaining a comprehensive grasp of root system fundamentals, integrating insights from root physiology, phenotyping and genetics, is a vital first step for engaging with advanced plant science. This foundational knowledge is paramount for developing sustainable agricultural practices, enhancing crop resilience and contributing to global food security in an era of environmental change.

## INTRODUCTION

Roots are foundational to terrestrial plant life and essential to global agriculture. In cultivated plants, root systems underpin plant productivity by absorbing water and nutrients, providing support and anchoring the plant in the soil, storing carbon, sequestering toxic substances, and mediating interactions with soil-dwelling organisms. Despite their critical importance, roots often receive less attention than shoots and reproductive organs regarding crop improvement and research, in part due to their hidden and less accessible nature. This Educational Review aims to bring these below-ground structures into the light, providing a clear entry point for understanding their complexity.

Cultivated plants have two main axes: the shoot and the root (Esau, 1965). A root is distinct from the shoot; it is defined as the portion of the plant that lacks leaves and nodes (the biologically active regions on shoots containing meristems). Physiologically, roots typically grow gravitropically, following the direction of gravity into the soil, and develop either from the radicle of the embryo or adventitiously from other meristematic regions (Crang *et al*., 2018). Although perhaps less immediately obvious than in above-ground vegetative and reproductive organs, roots too exhibit a wide range of morphologies and anatomical features, reflecting botanical diversity, their varied roles in the plant and supporting adaptation to different environments. In cultivated crops such as wheat (*Triticum aestivum*), maize (*Zea mays*), rice (*Oryza sativa*), common bean (*Phaseolus vulgaris*) and potatoes (*Solanum tuberosum*), root systems have been shaped by domestication and breeding to balance efficient nutrient acquisition with structural support, yield and environmental resilience (Smýkal *et al*., 2018).

Roots represent the primary interface between plants and the soil environment; their developmental behaviour, growth and functionality are deeply influenced by the physical, chemical and biological properties of the surrounding soil matrix (Lynch *et al*., 2012). In turn, root systems are increasingly being recognized as important active engineers of the soil environment, capable of influencing its physical, chemical and biological properties, and also affecting soil carbon dynamics and carbon sequestration (Fageria and Stone, 2006; Mueller *et al*., 2024). Roots act in this capacity through several mechanisms, largely through root growth and turnover, cell wall physical and chemical properties, and through production and release of exudates, and interaction with soil microbes (Wang *et al*., 2021). They also modulate soil structure through rhizodeposition, mechanical foraging and resource extraction; they mobilize and cycle nutrients through the soil; and serve as habitats for diverse microbial communities, which in turn are capable of generating new nutrient resources.

In both natural and managed ecosystems, the soil matrix is a highly heterogeneous environment, the properties of which vary significantly with depth, and over time (Vogel *et al*., 2022). Roots are capable of responding dynamically to environmental heterogeneity by altering the angle at which they grow through the soil (relative to the stem or older root tissue), lateral root branching patterns, root hair density, and anatomical features such as cortical aerenchyma or secondary cell wall thickening (Schneider and Lynch, 2020). Through this capacity for developmental response to the environment, roots can navigate through soil to grow towards and proliferate within nutrient-rich regions, limit growth in dry areas, avoid toxic compounds and waterlogged soil, and adapt to support the formation of beneficial symbioses, greatly enhancing plant productivity and survival in challenging environments (Karlova *et al*., 2021).

This review serves as a foundational guide on the biology of roots of cultivated plants, exploring what roots are and look like, the tools we use to understand them, and what determines their behaviour. We focus on cultivated plants due to experimental root research being frequently conducted in these species, and standardized terminology and conceptual frameworks for root systems are particularly important for communication of findings. This article emphasizes the structural and developmental foundations of root systems that underpin physiological function, introducing key physiological concepts where they intersect with anatomy, architecture and environmental response. It acts as a stepping stone for educators, students and researchers providing an overview of fundamental root functions and morphologies, highlighting root diversity and specializations, and underscoring the importance of understanding roots for sustainable crop production. A glossary of terms used throughout this review is provided in Box 1.

Box 1.
Glossary of terms: a summary of fundamental definitions regarding root morphology, anatomy, and physiology used to describe root systems in cultivated plants.

**Table:** 

Term	Definition
Adventitious roots	Roots that develop from non-root tissues, such as the stem (nodes), leaves, or modified structures like rhizomes and tubers.
Aerenchyma	Gas-filled spaces (lacunae) that form within the root cortex. These facilitate gas exchange (oxygen transport) in waterlogged conditions and reduce the metabolic cost of maintaining root tissue.
Allometry	The study of how the proportional size of body parts changes with overall body size.
Apparent plasticity	Also termed passive plasticity. A change in a trait that occurs in response to environmental conditions but lacks adaptive value, for example when root trait variation simply reflects allometric scaling with whole-plant growth or is a non-specific stress response that does not improve fitness.
Axial root	A collective term for the primary, basal and stem-borne roots (or ‘main roots’). These roots develop directly from the seed, stem or non-root plant body and serve as the major framework from which lateral roots branch.
Arbuscular mycorrhizal fungi (AMF)	A type of beneficial fungus that colonizes the root cortex, forming branched structures called arbuscules inside plant cells to exchange phosphorus and zinc for plant sugars.
Basal roots	Roots originating from the base of the hypocotyl or mesocotyl (the transition zone between shoot and root) in seedlings.
Brace roots	Aerial adventitious roots that emerge from stem nodes above the soil surface. They grow downwards to anchor the plant and are common in cereals like maize and sorghum. Also known as prop roots.
Cluster roots (proteoid roots)	Dense clusters of short lateral roots that form to increase surface area and exude organic acids, helping to mobilize phosphorus in nutrient-poor soils.
Contractile roots	Specialized roots found in geophytes (bulbs/corms) that shorten to pull the plant deeper into the soil for protection against temperature extremes.
Cortex	The tissue layer typically located between the epidermis and the stele. It functions in storage, transport and interaction with soil microbes.
Endodermis	The innermost layer of the cortex surrounding the stele. It develops barriers (like the Casparian strip) to regulate the selective flow of water and nutrients into the vascular system.
Epidermis	The outermost protective layer of the root, containing trichoblasts (hair-forming cells) and atrichoblasts, which serves as the primary interface for water and nutrient uptake.
Fibrous root system	A root system archetype common in monocots (e.g. grasses) characterized by many roots of similar size emerging from the stem, rather than a single dominant central root.
Geophyte	A plant that survives unfavourable seasons by means of underground storage organs such as bulbs, corms, tubers or rhizomes.
Gravitropism	The physiological growth response of roots to gravity, typically directing them to grow downwards into the soil.
Lateral roots	Roots that branch off from a parent root (an axial root or another lateral root). They are the primary sites for resource absorption.
Phenotypic plasticity	The ability of a single genotype to produce different physical traits (phenotypes) in response to different environmental conditions.
Primary root	The first root to emerge from a germinating seed, developing directly from the embryonic radicle.
Radicle	The embryonic root found inside the seed before germination.
Rhizodeposition	The process by which roots release organic compounds (exudates), cells and tissues into the surrounding soil, influencing soil chemistry and biology.
Root anatomy	The internal spatial and temporal arrangement of cells and tissues within a root (e.g. epidermis, cortex, stele), determining physiological functions like transport and barrier formation.
Root architecture	The spatial arrangement of the root system in the soil over time. It includes traits such as branching frequency, root angle, length and the overall distribution of roots.
Root economic space (RES)	A theoretical framework describing the trade-offs plants make in root investment, balancing resource conservation (thick, long-lived roots) vs. resource acquisition (thin, fast-growing roots).
Root hairs	Tubular extensions of epidermal cells (trichoblasts) that significantly increase the root surface area for water and nutrient absorption.
Stele	The central cylindrical core of the root containing the vascular tissues (including xylem and phloem).
Taproot system	A root system archetype common in dicots characterized by a dominant, central primary root (the taproot) from which smaller lateral roots branch.
Xylem	Vascular tissue within the stele responsible for transporting water and dissolved minerals from the roots to the shoot.

## FORM AND FUNCTION OF ROOTS IN CULTIVATED PLANTS

### Root physiology

Root physiology, the study of the function and mechanism of function of plant roots, encompasses the processes by which plant roots grow, take up resources, interact with their environment and contribute to whole-plant function. Root function is multifaceted, understood at various scales from the ecological roles of roots (Fitter, 2002), to physiological processes (Zobel, 1992), down to molecular mechanisms (Wang *et al*., 2007). Root physiology is a cornerstone of agricultural productivity, governing how effectively crops acquire water and nutrients, tolerate stress, and support shoot growth and development. Key physiological processes include the uptake and radial transport of water and dissolved ions through apoplastic and symplastic pathways, hormonal signalling that coordinates root growth and environmental responses, and the allocation of photosynthetically derived carbon between root growth, maintenance respiration and exudation. These processes are tightly coupled to root structure, operating within and constrained by the anatomical and architectural features described below.

### Root systems

**Fig. 1.**
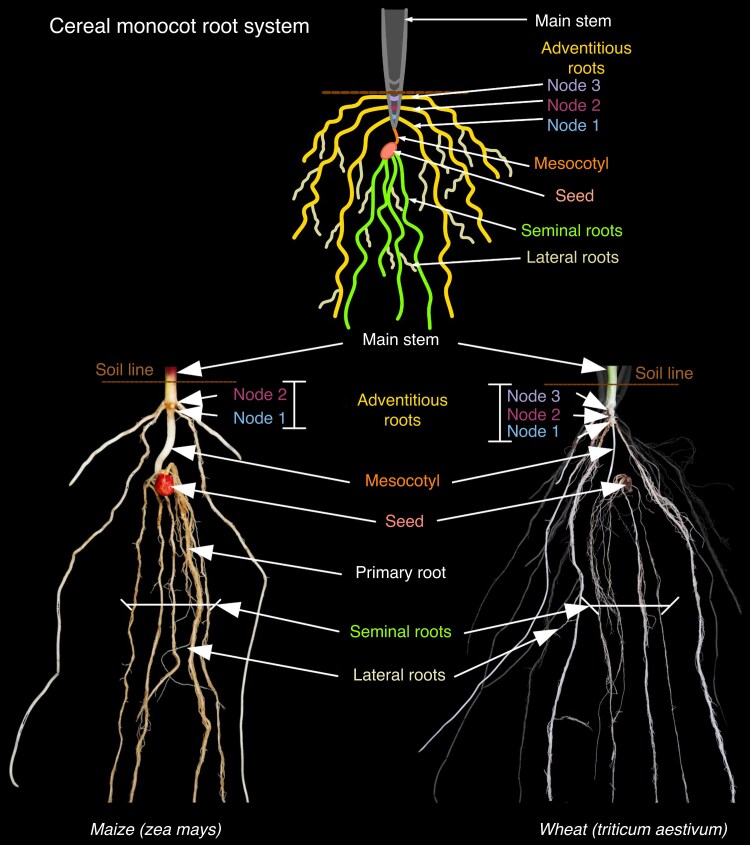
Photographic and schematic representations of cereal monocot root systems. The figure displays the root architecture of maize (*Zea mays*) and wheat (*Triticum aestivum*). Key root components, including seminal roots, crown/adventitious roots (nodal roots) and lateral roots, are annotated. Colours are used consistently across both the photographs and the schematic diagrams to facilitate visual comparison between the actual structures and their graphical representations.

**Fig. 2.**
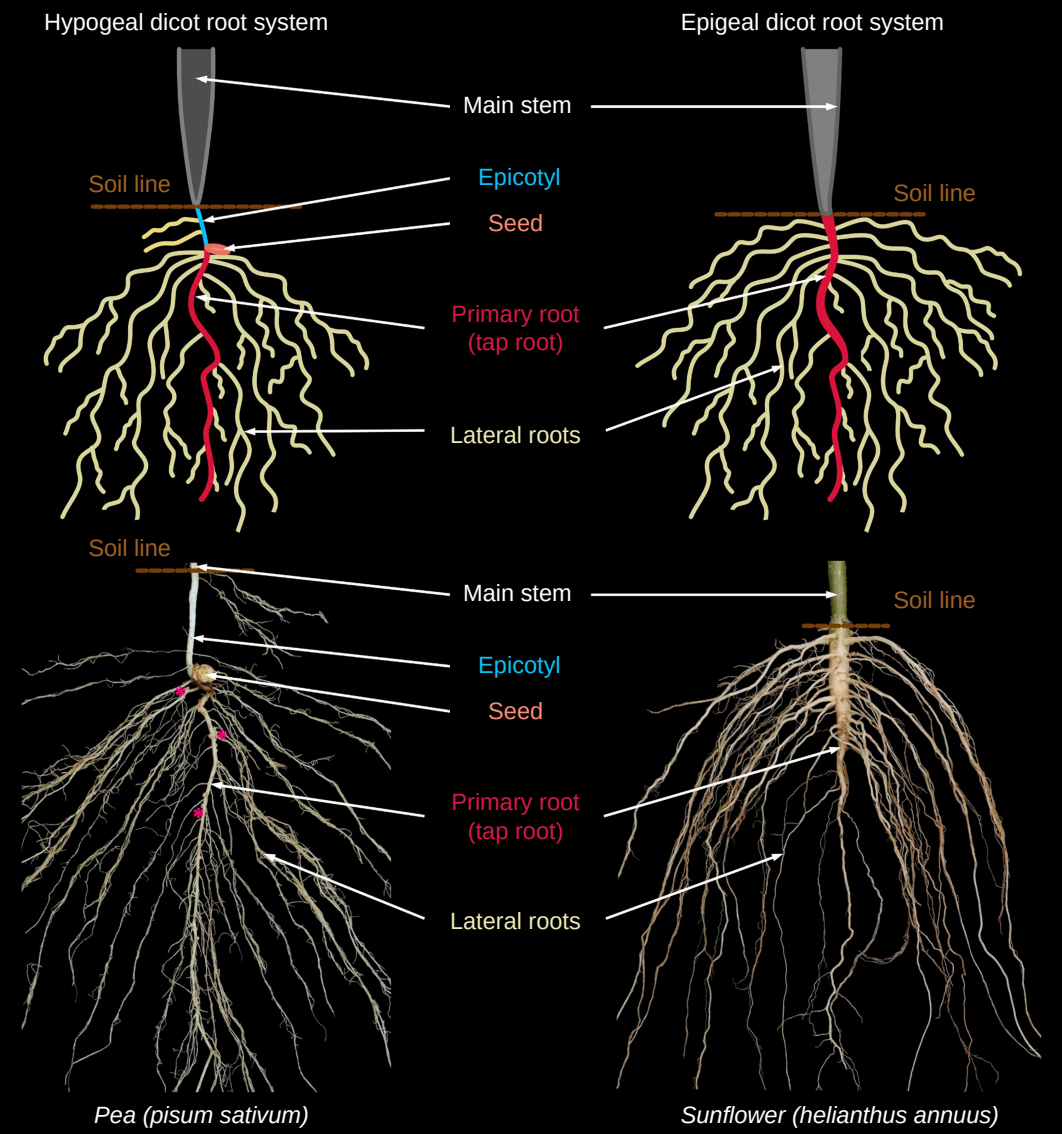
Photographic and schematic representations of dicot root systems. The figure contrasts the root morphology of a hypogeal germinating plant (pea, *Pisum sativum*) with that of an epigeal germinating plant (sunflower, *Helianthus annuus*). As in Fig. 1, individual root parts are colour-coded to allow for direct comparison between the images and illustrations. The asterisk (*) on the pea root system indicates the presence of root nodules formed by nitrogen-fixing bacteria.

Root systems, the total network of an individual plant’s roots, are broadly grouped into two major archetypes: taproot systems and fibrous root systems (Cannon, 1949; Li *et al*., 2025) (Figs 1 and 2). A taproot system features a dominant primary root, also known as the radicle, which grows gravitropically downwards to the nadir of the seed, undergoes secondary growth and gives rise to lateral roots. Further large roots can develop from stem tissue (described below), once the shoot is established (Fig. 2). This architecture is particular to dicotyledonous plants such as carrots (*Daucus carota*) (Zavadska *et al*., 2020), and common bean (Lynch and van Beem, 1993). In contrast, a fibrous root system typically consists of one or many embryonic roots, followed by flushes of adventitious roots emerging from stem tissue (Fig. 1). Fibrous root systems are found in monocotyledonous plants and ferns, such as wheat, onion (*Allium cepa*) and bracken (*Pteridium esculentum*) (Vande Kopple, 1975; Klepper, 1992; Hou and Blancaflor, 2009). In dicotyledonous plants, the cultivation system may affect the presentation of the root system; the taproot is the embryonic root and so when plants are vegetatively propagated, they lack a taproot and will produce adventitious roots resembling a fibrous root system. The roots within these systems are further classified or typified based on their developmental lineage, behaviour and function.

### Root classes

Despite their similar appearance, roots originating from different parts of the plant are not functionally identical, challenging the idea that their superficially common form dictates a singular function (Bushamuka and Zobel, 1998*a*, *b*; Waisel *et al*., 2002). While root classification has traditionally relied on morphological features (Zobel, 1975; Fahn, 1982), tissue of origin (Zobel, 1975), functional attributes [e.g. ecological function (Fitter, 2002; Freschet *et al*., 2021)] or hierarchical order-based attributes (Freschet *et al*., 2021) can also serve as a basis of root classification. Roots can originate from the shoot (shoot-borne), the seed (embryonic) or existing roots (root-borne). The biological distinctness of these origins was highlighted by the discovery of the maize *rt* (rootless) mutant by Jenkins (1930), which revealed a crucial difference in development and genetic control of formation between shoot-borne and root-borne roots. Several subsequent discoveries have been made, highlighting a complex and extensive underlying genetic control over root structural organization (Kirchoff *et al*., 2008), including how embryonic and shoot-borne roots are under distinct genetic control, as is lateral root production on these classes (Hochholdinger and Feix, 1998). These root class-specific genetic regulations in maize were reviewed in detail by Hochholdinger *et al.* (2018). Generally, four root classes and seven subclasses are widely used based on developmental origin (as reviewed in Zobel, 2011). However, defining root classes and subclasses has proven challenging and remains a central decades-old debate (Cannon, 1949; Weinhold, 1966; Böhm, 1979; Fitter, 1987; Pages and Kervella, 1990; Zobel, 2011; McCormack *et al*., 2015; Freschet *et al*., 2021).

The four developmentally delineated root classes are the primary, basal, stem-borne and lateral root (Zobel, 2011). The primary, basal and stem-borne are all mainly referred to as axial roots (or with varying terminology including primary root, main root, axile root and first-order root); these have the properties of developing directly from and being connected directly to the seed, stem or non-root plant body. Lateral roots have the distinction of being borne of root tissue, and are the ‘side’ branches of other roots. The class of lateral roots includes those borne from primary, basal and stem-borne roots, as well as those produced by other lateral roots. Further diversity beyond these classifications exists in Lycophytes, where roots branch dichotomously as opposed to laterally (Hetherington *et al*., 2020; Lakehal *et al*., 2023).

The primary root is the first to emerge from the seed, arising from the embryonic root axis (radicle), serving as the initial root system in many plants. In dicotyledonous plants, the primary root typically develops into the ‘taproot’ later in growth (Fig. 2). By contrast, in several monocots, seminal roots also develop from the seed and scutellar node, alongside the primary root, the number of which and the timing of their emergence vary between species and genotypes (Fig. 1). Collectively, these seed-borne roots are referred to as embryonic roots and comprise the embryonic root system.

Roots emerging from the region at the base of the taproot, below the cotyledons on the hypocotyl, epicotyl or mesocotyl [between the scutellar node and coleoptilar node (i.e. stem-like embryonic axis)], are considered the basal root class (Weinhold, 1966; Hoshikawa, 1969). Note that the terms ‘hypocotyl’ and ‘mesocotyl’ have a long-debated history in plant science, the consensus being that this tissue is neither shoot nor root (Esau, 1965; Fahn, 1982). In hypogeal germinators, roots emerging from the epicotyl are more specifically referred to as epicotyl roots. In epigeal germinators, roots emerging from the hypocotyl are referred to as hypocotyl roots, and those from the base of the hypocotyl as basal roots (Burridge *et al*., 2020).

Adventitious roots are those that develop from non-root tissues, such as the stem (main and tiller), modified stem structures such as rhizomes, stolons, tubers, bulbs, cladodes and corms. These roots can be classified based on their tissue of origin, as well as the position on the tissue of origin. In some monocotyledonous plants, such as brachypodium (*Brachypodium distachyon*), ‘coleoptile roots’ can emerge from the coleoptile node, the protective sheath covering the young shoot during germination (Watt *et al*., 2009). Further classification can be given based on the region of origin. In monocots, the production of stem-borne adventitious roots (i.e. nodal roots) is restricted to stem nodes; the basal-most stem nodes are typically highly compressed and produce many roots below ground (Steffens and Rasmussen, 2016). Roots produced underground are often referred to as crown roots, and those above the soil line as brace roots. These brace roots often show notable distinction to the rest of the root system, being thicker, having a highly developed hypodermis, pigmentation and altered root cap topology (Hostetler *et al*., 2021; Sparks, 2023). These are seen extensively in tropical grasses, such as maize, sorghum (*Sorghum bicolor*), sugarcane (*Saccharum officinarum*) and bamboo (*Bambusa vulgaris*), as well as in the Arecaceae (palm trees).

All of these described root classes can also develop lateral roots, roots developing from other root tissue (Bellini *et al*., 2014). Lateral roots can be hierarchically classified based on their branching position relative to the axial root, but conventions for assigning these root orders differ by discipline. In developmental and anatomical studies, the counting of the order of lateral roots begins with the first branching lateral (first-order) on the axial root, and subsequent branches to higher orders. However, in ecological studies, the most distal and finest root branches, with the most direct role in water and nutrient absorption, may be counted as the ‘first-order’ roots, increasing in order sequentially towards the base of the plant. These differing conventions can make comparing studies from across fields challenging (Freschet *et al*., 2021).

In well-characterized cereal crops, lateral roots can also be assigned ‘types’ based on their anatomy or growth rate, independent of their position in root architecture. This is best described in rice (*Oryza sativa*), where lateral roots are classified into L-type, which are able to branch, and are longer and thicker, or S-type, which are non-branching, shorter and thinner, with significantly different physiologies (Kawai *et al*., 2022). In pearl millet (*Pennisetum glaucum*), three lateral root types have been described based on growth rate and branching pattern, and with distinct anatomies (Passot *et al*., 2016, 2018). These different lateral root types are not exclusive to specific lateral root orders, though the formation of these different lateral root types is under developmental control and is affected by environmental conditions.

In classifying root systems, root hairs are often referred to as a ‘root type’, though this is a misnomer; root hairs are an extension of specific root epidermal cells. These are cellular features rather than an organ, and can form on all root types. Functionally, these tubular extensions are critical: they vastly expand the absorptive surface area for water and nutrients, aid in anchorage by adhering to soil particles, and serve as a primary interface for exudation and interaction with the rhizosphere microbiome (Rongsawat *et al*., 2021). The root epidermis is patterned with trichoblasts (root hair-producing cells) and atrichoblasts (non-root hair-producing cells) (Marzec *et al*., 2013). In cereals, these root hairs are arranged in longitudinal files, with trichoblasts alternating with atrichoblasts as a result of asymmetric cell division within a single epidermal cell file (Marzec *et al*., 2013). Dicotyledonous plants typically show other forms of root hair patterning, i.e. alternating around the circumference of the root as well as or rather than along individual cell files (Castillo-Jiménez *et al*., 2025). The proliferation of root hairs and their patterning is regulated and affected by many factors, including the positioning of the epidermal cell relative to underlying cortical cells and nutrient availability.

### Specialized roots

Beyond these developmental classifications, roots often develop with altered morphologies to perform specialized functions in their environment. While not necessarily developmentally distinct in origin, anthropogenically, we can typify these roots based on their function and context.

#### Storage roots

Storage roots are a common modification in cultivated plants, and can be seen in seminal and adventitious roots (Gregory and Wojciechowski, 2020). These are the product of extensive radial expansion, enabling this portion of root tissue to serve as reservoirs for carbohydrates, water or other essential nutrients. Taproot storage roots, exemplified by carrot and beet (*Beta vulgaris*), involve the hypertrophic thickening of the main axial root (Kuznetsova *et al*., 2020). Adventitious storage roots are also common, such as in cassava (*Manihot esculenta*) and sweet potato (*Ipomoea batatas*) (Sharma and Kaushal, 2016). These adventitious tuberous roots often show more variation in shape than the storage taproots.

#### Cluster roots *(proteoid roots)*

Cluster roots are root regions bearing extensive proliferation of short and densely packed lateral roots. These structures are well characterized for their exudation of organic acids (e.g. citrate and malate), which effectively chelate and mobilize nutrients with low solubility, particularly phosphorus, within nutrient-deficient soil environments (Neumann and Martinoia, 2002). Cluster roots are mostly observed in members of the family Proteaceae, such as lupin (*Lupinus albus*) (Watt and Evans, 1999).

#### Aerial roots

Many roots emerge or grow into the open air across a range of species and contexts, though they are often all labelled as ‘aerial roots’. This group can be broken down into functionally distinct subtypes. Brace roots (also termed prop roots), exemplified by cereals such as maize and sorghum, are robust adventitious roots that emerge from above-ground stem nodes. They typically grow downwards to anchor the plant, providing critical mechanical stabilization against high winds or lodging in tall, top-heavy species (Hostetler *et al*., 2021; Sparks, 2023). However, their role as primary mechanical stabilizers has been debated in some contexts, as brace roots presumably also contribute significantly to water and nutrient uptake (Hoppe *et al*., 1986; Wang *et al*., 1994). A related morphology, stilt roots, common in plants such as sugarcane and *Pandanus*, similarly originate from lower stem nodes but project obliquely, forming a broader basal support system, particularly advantageous in soft or waterlogged substrates (Ohira *et al*., 2013). In contrast, buttress roots are massive, plank-like extensions of superficial lateral roots at the base of large tropical trees. These structures enhance mechanical stability by broadly distributing the tree’s immense weight, thereby mitigating toppling, especially in nutrient-poor, shallow soils characteristic of rainforest ecosystems (Crook *et al*., 1997). Many cultivated and wild plants develop roots that grow in air, often with specific adaptations to this environment, though most function primarily for nutrient acquisition and mechanical stability. Stilt and brace roots frequently develop lenticels, small porous structures on their surface that facilitate gas exchange between internal root tissues and the atmosphere, which is particularly important for roots that traverse waterlogged or anaerobic zones before entering the soil (Barlow, 1994; Kawase, 1981).

#### Contractile roots

Predominantly observed in geophytes and certain monocots, such as those producing bulbs or fleshy rhizomes, contractile roots exhibit the unique ability to shorten, actively pulling the plant’s shoot or underground storage organs (e.g. bulbs, corms) deeper into the soil. This process optimally positions the plant for growth and provides essential protection against environmental extremes such as temperature fluctuations and desiccation (Pütz, 1992).

While this review focuses on cultivated plants, the diversity of root specializations across the plant kingdom is vast, and a comprehensive treatment of all root types is beyond its scope. A few examples serve to illustrate this breadth. Climbing roots, such as those of *Hedera helix* (ivy), are adventitious structures providing anchorage for vertical ascent on various substrates (Melzer *et al*., 2012). Within aerial environments, epiphytic plants have developed striking root specializations to survive without soil contact; hygroscopic roots, found in many orchids and some aroids, possess a multi-layered, spongy epidermis called the velamen, which efficiently absorbs and retains atmospheric moisture and nutrients (Ramesh *et al*., 2020). By contrast, certain aquatic species have lost many of the main ancestral functions of the root, but benefit from their presence mechanically and for orientation (Schutten *et al*., 2005). Other plants rely entirely on their root system for all resource acquisition, parasitizing other plants, or even fungi (*Monotropa uniflora*), through their roots (Leake, 1994). These examples illustrate that root specializations extend well beyond the forms typically encountered in cultivated systems, reflecting the broad adaptive capacity of roots across the plant kingdom.

## ROOT TRAITS AND ROOT PHENOTYPING

In biology, a phenotypic trait is a characteristic of an organism, the product of genetics and environment. ‘Trait’ derives from the Latin ‘tractus’, to draw or to drag, via the 16th-century French ‘trait’, referring to ‘the stroke of the pen or pencil in a picture’, and later coming to signify a characteristic. In the same sense, the combination and interaction of pen strokes form a picture; the combination of traits forms the phenotype.

Each trait itself is often a composite, made up of other measurable characteristics; for instance, root length is determined by cell number and cell length, which in turn may depend on cell wall thickness, division and expansion rate. Each trait often represents a network of other properties, both structural and dynamic, demanding a holistic approach to a comprehensive understanding of root physiology.

Our comprehension and characterization of plant phenotypic traits are inherently shaped by the available tools for observation and measurement. In their natural environment, most roots grow in soil, which, being opaque, makes it challenging to collect measurements without disturbance or while preserving sample integrity and context. Consequently, characterization of many root traits is intrinsically linked to the instruments and techniques used. Over-reliance on certain tools and methodologies leads to biases, termed the ‘law of the instrument’, and what can be measured becomes what is studied. To overcome this, we must develop and utilize diverse tools for root research, and in recent years this investigative toolkit has been expanded significantly, including high-resolution microscopy and high-throughput sectioning tools, 3D whole plant imaging and field-scale remote sensing of root activity.

Navigating the trade-off between root visibility and natural growth conditions requires the careful selection of cultivation systems. Hydroponic systems allow for continuous, non-destructive visualization of the root system and precise control over nutrient availability, though they lack the mechanical impedance and complex microbiome of natural soils. Moving towards more realistic substrates, glasshouse pot experiments introduce the soil matrix and controlled climatic conditions, yet they often restrict root spatial distribution due to container confinement and alter root density. Rhizotrons or rhizoboxes offer a compromise by enabling time-resolved monitoring of root system architecture in two dimensions against a transparent surface, though they still impose spatial constraints. In each instance, the ‘law of the instrument’ applies; for example, the exclusive use of rhizotrons or narrow pots may lead researchers to focus on 2D traits such as root depth, potentially overlooking complex 3D radial distribution patterns simply because the tool renders them invisible. Ultimately, to validate these observations in an agricultural context, field studies remain the gold standard. These investigations often rely on destructive methods, such as soil coring to quantify rooting depth and length density profiles, or manual excavation techniques to reveal architectural traits of the root crown, ensuring that phenotypes observed in controlled environments translate to the field. Non-destructive approaches, such as minirhizotrons, allow repeated observation of root growth and turnover *in situ* over time, complementing these destructive sampling methods (Odone and Thorup-Kristensen, 2025). Several recent articles have highlighted the latest technologies and tools that can be utilized for root phenotyping (Atkinson *et al*., 2019; Li *et al*., 2022; Jones *et al*., 2025*b*). As new tools emerge, this expands what we can observe and quantify, and enables us to relate functional significance to these new root traits, deepening our understanding of root physiology.

To organize these diverse measurements into a functional hierarchy, the concept of the root economic space (RES) has emerged. The RES is a multi-dimensional framework that describes how plants balance the carbon costs of constructing and maintaining roots against the returns in resource acquisition, positioning species along two orthogonal axes of variation (Pages and Kervella, 1990; Bergmann *et al*., 2020).

The first axis, the collaboration gradient, captures a trade-off in soil exploration strategies. At one end, ‘do-it-yourself’ plants invest carbon in constructing extensive, thin roots with high specific root length (SRL; root length per unit dry mass), enabling independent foraging. At the other end, ‘outsourcing’ plants produce thicker roots with greater cortical allocation to facilitate resource acquisition by symbiotic mycorrhizal fungi. Orthogonal to this, the conservation gradient reflects a trade-off between rapid returns and long-term investment. ‘Fast’ strategies are characterized by high nitrogen concentrations and metabolic activity, enabling rapid resource uptake but shorter root lifespans. In contrast, ‘slow’ strategies emphasize high root tissue density (RTD) and lignified tissues, enhancing protection and longevity. By integrating these axes with underlying anatomical and architectural traits, the RES provides a mechanistic framework linking root structure to function, enabling predictions of how specific traits, such as aerenchyma formation or secondary thickening, contribute to plant strategies for coping with environmental stress.

## ROOT ARCHITECTURE AND ANATOMY

Root architecture and anatomy are the two main overarching trait categories into which root system morphology is broken down to understand root physiology. Root architecture defines the arrangement of root organs and organ systems in the whole root system, and root anatomy refers to the arrangement of cells and tissues within these organs. Together these arch-traits largely determine root system physiology, and are targets for breeding for crop improvement and in understanding adaptation to diverse environments. Recently, an extensive handbook was developed to provide standardized methods and vocabularies to facilitate trait comparisons and data integration (Freschet *et al*., 2021). Here we provide a general overview.

### Root architecture

Root system architecture describes the arrangement of the root system in space and time. This includes traits such as the number, length and surface area of roots, number of root-producing stem nodes, lateral root branching frequency and length, as well as root angle and position. Together these describe the volume and extent of soil exploration and therefore the capacity of the plant for direct nutrient capture (Figs 1 and 2).

As roots are largely immobile once developed, their spatial development has a lasting significant effect on plant performance. Different resources are available in different regions within the soil, and growth to and within these regions is the plant’s only way of accessing these resources; therefore, the benefits of different root system architectural configurations in resource capture are also highly context dependent. This is similarly true in providing anchorage and support to the plant shoot; different rooting rates, height or extent of root production, and root angle, will affect the stability of the stem, though how necessary support is also depends on the environment in which the plant is grown.

Across the flowering plants, there are broadly conserved themes in root architectural development, such as fibrous and taproot systems. In annual monocotyledonous crops, the root system develops in a distinct pattern; the primary root emerges from the germinating seed, followed by seminal roots, and subsequently, successive layers of adventitious nodal roots that arise from stem nodes near or above the soil surface throughout the plant’s vegetative growth (Klepper, 1992). In contrast, annual dicotyledonous crops typically establish a scaffold of axial roots, comprising a primary root and other dominant axial roots, soon after germination. This initial framework largely determines the overall architecture and early soil exploration capacity of the seedling root system (Strock *et al*., 2019). In both monocotyledonous and dicotyledonous plants, lateral roots collectively contribute the majority of the total length of the root system and are crucial sites for nutrient acquisition and symbiotic associations with mycorrhizal fungi (Brundrett *et al*., 1996; Guo *et al*., 2008; Smith and Read, 2008), though their distribution is ultimately determined by the axial roots from which they branch. Across the root system, these distinct root classes and subclasses possess varying efficiencies for nutrient and water uptake and transport, largely determined by their cellular anatomy (Ahmed *et al*., 2018; Schneider *et al*., 2020*b*).

### Root anatomy

**Fig. 3.**
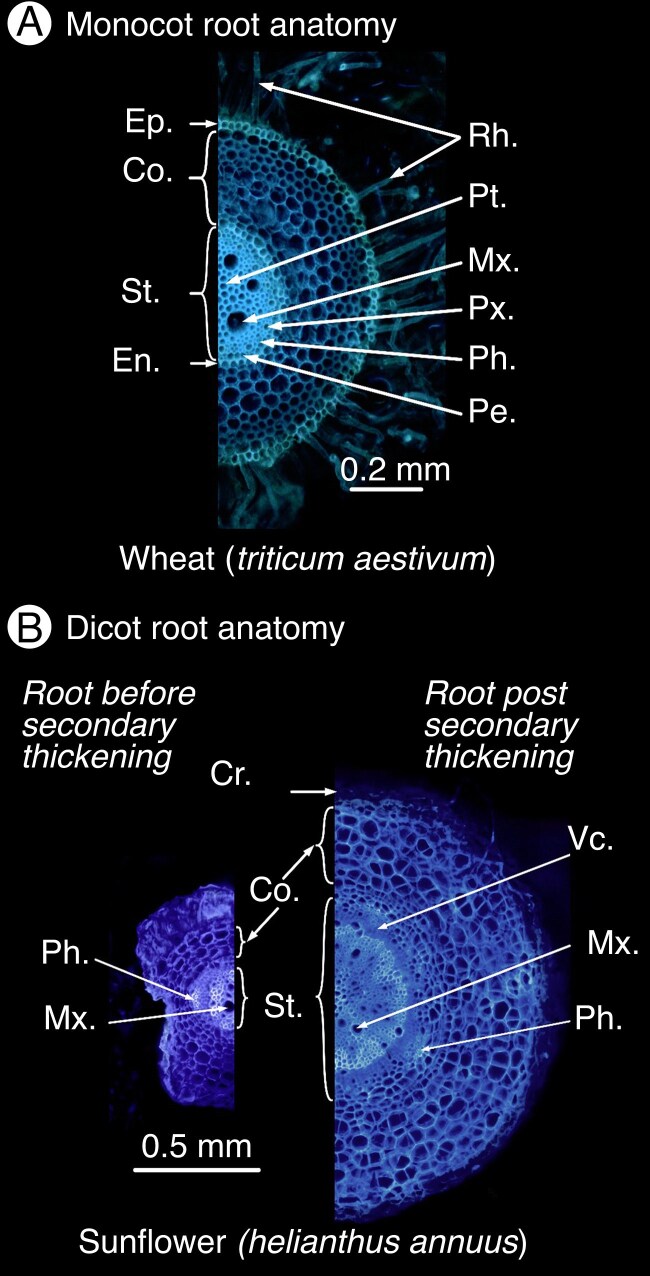
Transverse sections showing the anatomy of monocot and dicot roots. (A) Anatomy of a monocot root (wheat, *Triticum aestivum*). (B) Anatomy of a dicot root (sunflower, *Helianthus annuus*), showing the root structure before secondary thickening (left) and post-secondary thickening (right). Abbreviations: Co, cortex; Cr, cork; En, endodermis; Ep, epidermis; Mx, metaxylem; Pe, pericycle; Ph, phloem; Pt, pth; Px, protoxylem; Rh, root hair; St, stele; Vc, vascular cambium.

Root (cellular) anatomy refers to the spatial and temporal arrangement of tissues and cells within the root (Lynch *et al*., 2021) (Fig. 3). Roots generally exhibit a high degree of radial symmetry, and so their anatomy is typically examined in both longitudinal and transverse sections. Longitudinal sections are particularly informative near the root tip, where cells are actively dividing and elongating, as this enables visualization of cell file trajectories, cell length, patterns in cell wall modification and lateral root primordia. Transverse sections are often used in more mature regions as they allow for visualization of the full complement of tissue and cell types present at a certain position and quantification of traits that allow investigation of functional properties (Baca Cabrera *et al*., 2026).

Longitudinally and transversely, the root tip is divided into distinct zones. Longitudinally, we assign these zones based on the different stages of root cell development; as cells age while the root grows they progress from the most nascent dividing cells at the root tip apex (division zone), to the elongation zone where cells elongate and push the root tip forward, to the zone of maturation where cell wall thickening and root hair production occur (Ishikawa and Evans, 1995).

Transversely, the root is divided into three primary tissue layers: the epidermis (outermost), cortex, also widely referred to as ground tissue (middle), and stele (centre). The epidermis is composed of trichoblasts (root hair-bearing cells) and atrichoblasts (non-root hair-bearing cells), and is the layer of the root in direct contact with and responsible for interaction with the environment, as well as nutrient uptake. The cortex has many functions, including mediating nutrient transport, production and storage of metabolites, and hosting microbial interactions. The cortex comprises several varied cell types and tissues that contribute to these functions, including the endodermis, cortical parenchyma, aerenchyma, sclerenchyma and hypodermis. Summarized briefly, the endodermis is the innermost layer of the cortex immediately bounding the stele. This layer develops extensive cell wall secondary thickening in order to regulate the movement of water, gases and nutrients between the cortex and the stele. The cortical parenchymal cells produce metabolites, host interaction with symbionts such as arbuscular mycorrhizal fungi and bacteria, and transport water and nutrients from the epidermis to the stele. Aerenchyma are gas-filled lacunae that develop in the cortex, both through cell patterning and programmed cell death; these enable gas diffusion and reduce root metabolic activity. Sclerenchyma are cells with specially thickened cell walls, which can either be distributed throughout the cortex, often around aerenchyma, or in layer(s) in the outer cortex (*Jones et al., 2025a*; Kawa *et al*., 2025). The exodermis is a tissue layer in the outer region of the cortex, often used interchangeably with hypodermis, though the hypodermis can include several layers of the outer cortex and is positionally defined, and the exodermis is defined by the development of Casparian bands and suberin lamellae in the cell walls of this layer. The exodermis functions as a second barrier layer to the movement of water and nutrients, and provides mechanical strength (Lynch *et al*., 2021; Jones *et al*., 2025*a*). The stele, also referred to as the vascular cylinder, is the central vascular column of the root, containing phloem and xylem (including the enlarged metaxylem) vessels, responsible for transporting water and nutrients from the root to the shoot, and photoassimilates from the shoot to the root. The outermost cell layer of the stele is the pericycle, a meristematic tissue from which lateral root primordia originate and which, in dicots, contributes to the vascular cambium during secondary growth.

This configuration is common across all vascular plants, though the arrangement of cell types in these layers differs between different plant varieties, species, and dramatically between dicots and monocots. In dicots, the stele typically has a xylem pole arrangement with a specific number of xylem poles. Monocot roots, in contrast, have a vascular arrangement with many xylem and phloem strands organized around a large central pith, a feature typically absent in dicot roots (Tomlinson, 1970). Additionally, monocots often form a well-developed exodermis, a secondary protective layer just beneath the epidermis, important for regulating water loss and pathogen entry (Liu and Kreszies, 2023). Dicot roots are often characterized by having a vascular cambium (lateral meristem) that produces new xylem and phloem continuously through secondary growth, thereby destroying primary tissues such as the epidermis, cortex and endodermis (Ragni and Greb, 2018).

## EFFECTORS OF ROOT PHYSIOLOGY

### Plasticity

**Fig. 4.**
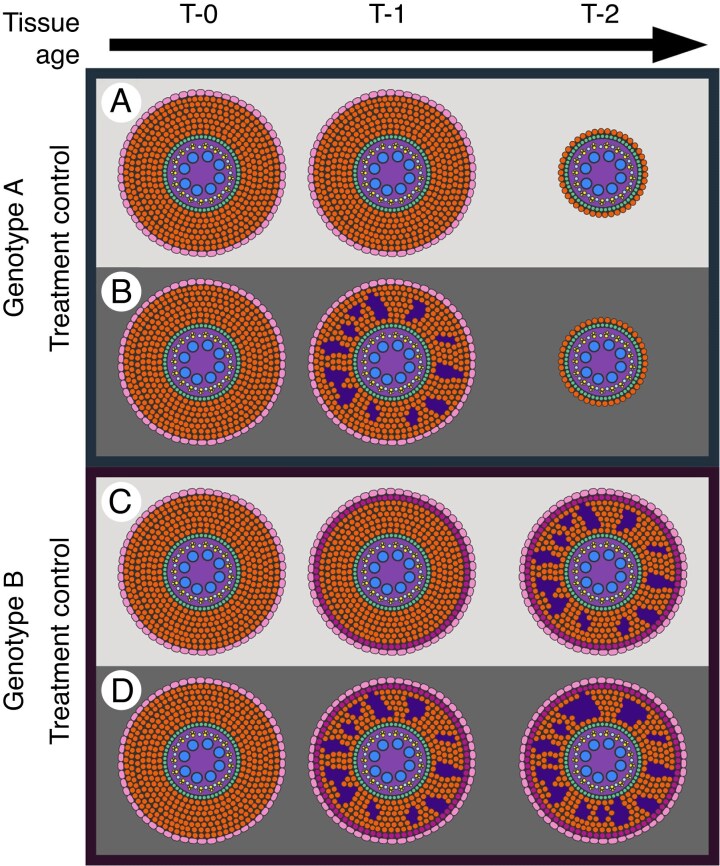
Root anatomical plasticity in cereal crop genotypes under stress. The illustrations depict root cross-sections of two hypothetical genotypes (A and B) across three timepoints: T-0 (shortly after root development), T-1 (after stress imposition and response) and T-2 (plant maturity). Panels (A) and (B) represent Genotype A. This genotype shows a trajectory toward complete cortical senescence. While a plastic response (formation of root cortical aerenchyma) is visible in the treated plants at T-1, extensive cortical senescence at maturity (T-2), common to both control and treated plants, masks these earlier anatomical differences. Panels (C) and (D) represent Genotype B. By T-1, hypodermal secondary thickening has occurred. In treated plants, this is accompanied by the formation of aerenchyma lacunae. By T-2, both treated and control plants have developed aerenchyma lacunae; however, the trait remains more pronounced in the treated plants, although the distinction is less obvious than at T-1. Tissue colour key: epidermis (pink), hypodermis (violet), cortical parenchyma (amber), cortical aerenchyma (indigo), endodermis (green).

The phenotype (P) is the physical expression of the plant’s genome, and is a product of the interaction of this genetic information (G) with the plant’s growth environment (E), i.e. P = G × E. It can also be calculated as a product of interaction between the above factors and management practices (M), i.e. P = G × E × M. The ability of a single genotype to produce different phenotypes in response to environmental conditions is known as phenotypic plasticity. The effects of environmental interactions can be major fundamental developmental differences, altered growth dynamics and organ size, or a change in senescence patterns. The phenotyping of plasticity in many traits will depend on the timing of data collection, as an effect of the environment on a trait value may be masked by later developmental processes. For example, a root may develop a larger number of cortical cell file layers in response to compaction, and then the root cortex may senesce to the same extent as a root in a non-compacted environment, leaving little indication of the earlier plastic response at this scale. These more transient plastic phenotypes, often related to temporary growth inhibition, have been called elastic or reversibly plastic as subcategories of plastic responses (Colombi *et al*., 2024); both of these indicate the criticality of timing of phenotyping in understanding the role of plasticity in root physiology (Fig. 4).

Plasticity can change plant fitness positively, negatively or neutrally depending on the cultivar and the environment. In the context of roots and soil, plasticity can enable a single genotype to express different root traits and combinations of traits that enable the root system to acquire resources efficiently across a range of environments. As the soil environment can be complex, heterogeneous and dynamic in its resource availability, the ability to produce different root phenotypes in response to varied or changing conditions can be highly beneficial (Schneider and Lynch, 2020). Where this environmental response produces a root phenotype with a contextual benefit to plant fitness, this is an adaptive response. However, not all plasticity is innately adaptive; ‘apparent plasticity’ (i.e. passive plasticity), which lacks adaptive value, can also occur, such as in cases where the response is allometric with whole plant development, or specific stress responses that do not affect fitness. Plasticity can also be maladaptive, typically arising as a result of environmental responses that may have had adaptive value in an ancestral context, but is counterproductive in managed or agricultural ecosystems (Schneider, 2022). Root trait plasticity may be significantly related to yield and yield stability under altered environmental conditions (Zhao *et al*., 2025), and the regulation of these plastic responses has been shown to be under genetic control (Schneider, *et al*., 2020*a*), paving the way for consideration of plasticity as a desirable breeding target.

### Abiotic environmental interaction

Roots demonstrate remarkable plasticity in their anatomy and architecture, a trait observed across numerous plant species and diverse environmental conditions. This adaptability allows plants to optimize resource acquisition and enhance survival, particularly under stress. For instance, studies on soybean (*Glycine max*) subjected to drought revealed an increase in metaxylem vessel number, which boosts the root’s ability to conduct water. Simultaneously, a reduction in total cortical area was observed, decreasing the metabolic energy needed to access deep soil water (Prince *et al*., 2017). Similarly, rice grown under drought and low phosphorus conditions exhibited greater plasticity in root architectural traits, correlating with improved yield stability (Sandhu *et al*., 2016).

Further observations in rice under water stress have highlighted the adaptive nature of root plasticity, with changes in root length and root cortical aerenchyma formation leading to greater shoot biomass and yield (Niones *et al*., 2012, 2013). In both wheat and rice experiencing water deficit, xylem vessel diameter and number, along with stele diameter, showed significant plasticity (Kadam *et al*., 2017). Beyond these specific examples, various root traits show plastic responses to water deficit. These include the number of nodal roots in rice (Suralta *et al*., 2010) and maize (Gao and Lynch, 2016), lateral branching density and length in maize (Zhan *et al*., 2015), and deep rooting in wheat (Ehdaie *et al*., 2012; Wasson *et al*., 2012), millet (Rostamza *et al*., 2013), rice (Hazman and Brown, 2018) and maize (Nakamoto, 1993). Plasticity to multiple integrated root phenotypes in maize was associated with drought tolerance (Klein *et al*., 2020).

In common bean, plasticity in secondary root growth has been linked to variations in root depth and shoot growth, particularly in low phosphorus environments (Strock *et al*., 2018). Rice also demonstrates plasticity in lateral root length and density (Kano *et al*., 2011; Kano-Nakata *et al*., 2013), as well as root length density and total root length (Kano-Nakata *et al*., 2011; Tran *et al*., 2015). These adaptations have been associated with increased shoot biomass, water uptake and photosynthetic efficiency during drought.

Nutrient availability also triggers plastic root responses. For example, barley (*Hordeum vulgare*) exhibits lateral root proliferation when exposed to nitrogen-rich patches (Drew, 1975), and maize shows similar responses to phosphorus patches (Yano and Kume, 2005). These plastic responses can be root class specific or manifest more clearly in one root class than another. Hydropatterning, a specific plastic response, involves the directed development of lateral branches, root hairs and aerenchyma towards available water (Bao *et al*., 2014). Even root hair morphology can be plastic; maize genotypes that developed longer root hairs under low phosphorus conditions outperformed those with consistently long root hairs (Zhu *et al*., 2010).

In summary, root anatomical and architectural characteristics display a wide array of plastic responses across diverse environments. However, a comprehensive understanding of which specific plastic responses are truly adaptive and how different root traits interact to confer adaptive advantages under edaphic stress remains an active area of research.

### Biotic environmental interaction

Roots are dynamic interfaces between plants and their soil or aerial environments, where they engage in complex interactions with biotic and abiotic factors. These interactions include mutualistic associations with microbes, defence responses to pathogens and parasites, and morphological adaptations to challenging environments. Such root–environment relationships play a critical role in shaping plant fitness, nutrient acquisition and ecosystem-level processes. Here, we describe a number of modifications of roots driven by their biotic environment in cultivated plants.

#### Mycorrhizal associations

Roots frequently establish mutualistic associations with diverse fungal partners. Among the most widespread are arbuscular mycorrhizal fungi (AMF), which colonize the host root cortex, forming complex, branched arbuscules within cortical cells. This intercellular fungal network facilitates a pivotal bidirectional exchange: plant-derived carbohydrates are transferred to the fungus in return for enhanced acquisition of primarily soil-immobile nutrients, notably phosphorus and zinc (Lanfranco *et al*., 2016). Interestingly, the configuration of root anatomy can impact colonization of AMF, as aerenchyma lacunae can significantly reduce cortical habitat for colonization (Galindo-Castañeda *et al*., 2019). Other major types of mycorrhizae include ectomycorrhizae, which are predominantly found in woody species and are characterized by the formation of a dense fungal mantle around the root tip and an intercellular Hartig net within the cortex (Martin *et al*., 2016). These ubiquitous fungal symbioses fundamentally augment nutrient acquisition efficiency, confer enhanced drought tolerance and bolster pathogen defence mechanisms, underscoring their integral role in root functionality across both natural and anthropogenically managed ecosystems.

#### Nodulated *r*oots

Nodulated roots represent a highly specialized interaction with nitrogen-fixing bacteria, notably *Rhizobium* and *Bradyrhizobium* species in legumes and certain non-leguminous taxa (e.g. *Parasponia*) (Franche *et al*., 2009). These anatomically distinct root structures internally accommodate differentiated bacterial cells, termed bacteroids, which catalyse the energetically demanding conversion of atmospheric dinitrogen (N_2_) into bioavailable ammonia through the action of the nitrogenase enzyme complex. The complex developmental orchestration of these nodules is under stringent regulation by both conserved plant genetic pathways, such as the Nod factor signalling cascade, and environmental cues, particularly edaphic nitrogen availability (Ferguson and Mathesius, 2003). These symbiotic organs are indispensable for the global nitrogen cycle, contributing substantially to both natural ecosystem fertility and sustainable agricultural productivity.

#### Root-knot nematodes

Roots are also susceptible to parasitic interactions with plant pathogens, exemplified by root-knot nematodes (*Meloidogyne* spp.). Upon invasion of root tissue, these endoparasites induce pronounced developmental aberrations, leading to the formation of characteristic galls or ‘knots’ through localized hypertrophy and hyperplasia of host root cells. Within these induced galls, nematodes establish specialized feeding structures known as giant cells, which effectively act as metabolic sinks, re-directing host nutrients and severely disrupting normal root physiological processes, frequently resulting in significant reductions in plant growth and agricultural yield (Rutter *et al*., 2022).

#### Haustorial roots

Exclusive to parasitic plants, such as *Cuscuta* (dodder) and *Striga* (witchweed), haustorial roots represent highly specialized intrusive organs. These structures are designed to penetrate the vascular tissues of host plants, establishing direct physiological conduits for the direct extraction of water, nutrients and photosynthates. Haustoria can form anatomical connections with both the xylem and phloem of the host, thereby circumventing the conventional edaphic absorption mechanisms. The obligate or facultative nature of the parasitic lifestyle (e.g. *Striga* species being obligate versus some *Triphysaria* species being facultative) directly influences haustorium development, host specificity and the phenology of host attachment (Yoshida *et al*., 2016). Similarly to the colonization of AMF, root anatomy can alter *Striga* infection (Kawa *et al*., 2024).

These diverse root–environment interactions, ranging from beneficial mutualisms to detrimental parasitism, fundamentally dictate a plant’s ability to thrive. Understanding the intricate molecular and physiological mechanisms underpinning these relationships is therefore crucial, not only for deciphering plant ecological strategies but also for developing sustainable agricultural practices that utilize beneficial symbioses and mitigate the impact of root pathogens.

## GENETIC CONTROL OF ROOT TRAITS

Compared to above-ground traits, fewer genetic loci have been identified for root architectural and anatomical features. Nevertheless, genomic research has identified several promising candidate genes crucial for modulating desirable root traits. For instance, in rice, the *DRO1* gene has been linked to deeper rooting, leading to enhanced nitrogen (Arai-Sanoh *et al*., 2014) and water uptake (Uga *et al*., 2013), and ultimately higher yields. More recently, the gene *CIPK15* in maize was found to correlate with a steeper nodal root angle, contributing to improved nitrogen acquisition and increased shoot biomass in nitrogen-deficient conditions (Schneider *et al*., 2022). Beyond nutrient uptake, genes such as *EGT2* and *EGT1* in wheat and barley play a pivotal role in regulating root angle in response to gravity (Kirschner *et al*., 2021; Fusi *et al*., 2022). The transcription factor bHLH121 regulates root cortical aerenchyma formation in maize (Schneider *et al*., 2023).

In the context of phosphorus acquisition, *PSTOL1* in rice promotes early root growth, boosting phosphorus uptake and grain yield in low-phosphorus soils (Gamuyao *et al*., 2012), while *JAZ11* enhances primary and seminal root elongation, thereby improving phosphorus foraging (Pandey *et al*., 2021). Furthermore, various transcription factor families, including ethylene response factors (ERFs), dehydration-responsive element binding (DREB) proteins, zinc finger proteins (ZFPs), WRKY and MYB, have been identified as key regulators, both positive and negative, of drought responses across species such as wheat, maize, rice and *Arabidopsis* (Ergen *et al*., 2009; Kulkarni *et al*., 2017).

Significantly, recent research has demonstrated that root plasticity is genetically controlled, with distinct genetic loci governing plastic responses compared to those controlling trait expression under optimal conditions (*Schneider et al., 2020b*, *c*). Consequently, uncovering the genetic control of root traits demands careful consideration of plant age and the growing environment. Seedling root systems or plants grown in artificial media may be poor predictors of mature, field-grown root systems (Zhu *et al*., 2011) and can even be under different genetic control (Hochholdinger *et al*., 2004). Therefore, genetic studies on mature plants in relevant environments are vital for a comprehensive understanding of the genetic architecture of root traits and enhancing nutrient efficiency.

These ongoing genomic discoveries are rapidly expanding our understanding of the genetic architecture underlying root development and function. The identification of such key genes and regulatory networks offers immense potential for targeted breeding strategies and genetic engineering, ultimately enabling the development of crop varieties with optimized root systems for enhanced resource use efficiency and improved resilience in the face of challenging environmental conditions.

However, breeding for root traits does come with challenges. Genetic mapping studies show that both root anatomical and architectural traits are heritable, yet they are typically quantitative characteristics controlled by many genes with small effects. This polygenic nature complicates traditional single-trait breeding strategies, making it impractical to stack hundreds of genes in an ideotype breeding approach. However, genomic selection offers a promising tool for selecting multiple loci simultaneously. These methods should consider not only individual root traits but also their integrated plastic responses. Including wild germplasm and landraces in training sets is crucial, as elite crops, developed in high-input environments, may have lost beneficial root plasticity or foraging strategies suited for low-input conditions. Wild accessions therefore offer a broader range of phenotypic variation and plasticity in root traits.

Another complication is the probable pleiotropic nature of many genes controlling these traits. For instance, ethylene, a common signalling mechanism, regulates diverse root traits such as elongation, programmed cell death (e.g. aerenchyma formation and cortical senescence) (Yamauchi *et al*., 2016; Schneider *et al*., 2018), and lateral root development (Negi *et al*., 2008). While upregulating ethylene-related genes might enhance a beneficial trait in one environment, it could unintentionally impact other traits, leading to maladaptive outcomes. Additionally, a few recent studies suggest that genes associated with root traits also pleiotropically act with above-ground shoot traits in wheat and sorghum (Li *et al*., 2019; Kebede *et al*., 2025). For example, in wheat, some loci associated with root depth were also linked to shoot traits, such as canopy temperature and grain yield per plant, indicating a genetic connection between root and shoot development (Li *et al*., 2019).

## ROOTS IN SUSTAINABLE AGRICULTURE

In the face of a rapidly changing climate, characterized by more frequent and intense droughts, floods and nutrient limitations, research into plant roots is essential for ensuring sustainable agriculture. Roots are the primary interface between plants and the soil, responsible for vital functions such as water and nutrient uptake, anchoring, and critical interactions with the soil microbiome. Understanding and optimizing root system architecture and function can dramatically enhance crop resilience, enabling plants to access deeper water reserves, efficiently acquire scarce nutrients, and withstand physical stresses such as erosion or mechanical impedance. Furthermore, deeper and more robust root systems play a critical role in carbon sequestration, which involves moving atmospheric carbon into stable soil pools, thereby contributing to climate change mitigation. By studying the genetic and physiological mechanisms that govern root development and their responses to environmental cues, researchers can inform breeding programmes and agricultural practices to cultivate crops that are not only productive, but also inherently better adapted to the challenges of future agroecosystems, ultimately bolstering global food security.

The global agricultural landscape urgently requires crop varieties capable of withstanding edaphic stress. While essential for all land plants, root architecture and anatomy hold particular importance for crops, given their intensive cultivation and direct contribution to human welfare, unlike less managed ecosystems such as forests and pastures. Thus, it is imperative to utilize existing genetic variation to cultivate root systems that excel at acquiring and utilizing soil resources. Practically, this can be achieved through genomic selection programmes that incorporate root trait phenotyping data from field conditions, by screening diverse germplasm collections including landraces and wild relatives for beneficial root characteristics, and by integrating root ideotype targets into existing breeding pipelines alongside shoot and yield traits. Advances in high-throughput root phenotyping and the identification of genetic loci controlling root traits, as described above, are making these approaches increasingly feasible.

## CONCLUSIONS

### Promising avenues in root research

Looking ahead, several research avenues show particular promise for advancing our understanding of roots in cultivated plants. Continued advancements in root phenotyping technologies, especially those enabling non-destructive, high-throughput imaging in complex soil environments, are critical for bridging the gap between controlled laboratory observations and field conditions. Further exploration into the genetic control of root traits, utilizing mutation analysis and genomic tools, will be instrumental in identifying and manipulating key genes that confer advantages in resource acquisition and stress tolerance. Deeper investigation into the plasticity of root system architecture in response to varying environmental cues, opportunities and stresses can inform breeding strategies for more adaptable crops. Finally, a more comprehensive understanding of root–microbe interactions within the rhizosphere, including the complex dynamics of nodulated roots and diverse mycorrhizal associations, will be essential for developing sustainable agricultural practices that utilize natural biological processes for enhanced plant performance.

## SUMMARY

Roots are indispensable yet often underappreciated organs that form the foundation of plant life and agricultural productivity. This review has elucidated the complexity and indispensable nature of root systems in cultivated plants, highlighting their multifaceted roles from fundamental anchorage and resource acquisition to complex biotic interactions. We have explored the diverse morphologies and anatomies that define root classes and specialized types, recognizing that what roots ‘look like’ is inextricably linked to ‘what they do’ and ‘how they function’ across various scales. The nuanced interplay of root physiology, encompassing everything from subcellular ion transport to whole-plant resource allocation and environmental responsiveness, underscores the profound plasticity that enables roots to adapt to heterogeneous and often challenging subterranean conditions.

Crucially, the inherent inaccessibility of the root system has historically presented significant challenges to comprehensive characterization. However, advancements in root phenotyping methodologies, coupled with sophisticated genetic analyses, are progressively dismantling these barriers. The application of mutation-based studies, particularly in model crops such as maize, continues to refine our understanding of genetically distinct root classes, revealing a structural and developmental ontology far more intricate than previously conceived.

The insights garnered from studying root architecture and anatomy, and specialized root functions, alongside their genetic and environmental effectors, are not merely academic pursuits. They are instrumental for advancing sustainable agricultural practices and breeding resilient crop varieties. By deepening our understanding of how roots acquire essential resources, withstand diverse stressors and engage with the complex soil microbiome, we can strategically optimize root architectural and anatomical traits for enhanced productivity, reduced environmental footprint and improved food security in an era of escalating global demands and climate variability. Continued interdisciplinary research into this hidden half of the plant promises to unlock opportunities for agricultural innovation, offering a fertile ground for the next generation of plant scientists to make global impacts.
